# Repurposing Cationic Amphiphilic Drugs and Derivatives to Engage Lysosomal Cell Death in Cancer Treatment

**DOI:** 10.3389/fonc.2020.605361

**Published:** 2020-12-10

**Authors:** Michelle Hu, Kermit L. Carraway

**Affiliations:** ^1^Department of Biochemistry and Molecular Medicine, UC Davis School of Medicine, Sacramento, CA, United States; ^2^UC Davis Comprehensive Cancer Center, UC Davis School of Medicine, Sacramento, CA, United States

**Keywords:** cancer treatment, therapeutic resistance, therapeutic targeting, therapeutic repurposing, necrosis, lysosomal cell death, lysosomal membrane permeabilization, cationic amphiphilic drugs

## Abstract

A major confounding issue in the successful treatment of cancer is the existence of tumor cell populations that resist therapeutic agents and regimens. While tremendous effort has gone into understanding the biochemical mechanisms underlying resistance to each traditional and targeted therapeutic, a broader approach to the problem may emerge from the recognition that existing anti-cancer agents elicit their cytotoxic effects almost exclusively through apoptosis. Considering the myriad mechanisms cancer cells employ to subvert apoptotic death, an attractive alternative approach would leverage programmed necrotic mechanisms to side-step therapeutic resistance to apoptosis-inducing agents. Lysosomal cell death (LCD) is a programmed necrotic cell death mechanism that is engaged upon the compromise of the limiting membrane of the lysosome, a process called lysosomal membrane permeabilization (LMP). The release of lysosomal components into the cytosol upon LMP triggers biochemical cascades that lead to plasma membrane rupture and necrotic cell death. Interestingly, the process of cellular transformation appears to render the limiting lysosomal membranes of tumor cells more fragile than non-transformed cells, offering a potential therapeutic window for drug development. Here we outline the concepts of LMP and LCD, and discuss strategies for the development of agents to engage these processes. Importantly, the potential exists for existing cationic amphiphilic drugs such as antidepressants, antibiotics, antiarrhythmics, and diuretics to be repurposed to engage LCD within therapy-resistant tumor cell populations.

## Introduction

Despite decades of research into its underlying drivers and the development of corresponding therapeutic agents, cancer remains the second leading cause of death in the United States. Moreover, worldwide cancer incidence and death rates are predicted to increase by two-thirds over the next two decades as a result of an expanding and aging population (1). A potential barrier to therapeutic outcomes concerns the specific cytotoxic mechanism by which anti-cancer agents act. The overwhelming majority of conventional and targeted cancer therapeutics employed in the clinic today kill tumor cells *via* caspase-dependent apoptosis, characterized by the breakdown of cellular components and their distribution into apoptotic bodies that are consumed by phagocytic cells (2). However, suppression of apoptosis is a hallmark of cancer (3); cancer cells engage a variety of strategies to subvert apoptotic mechanisms and engage anti-apoptotic pathways to promote their expansion, therapeutic resistance, and progression to malignancy. These general observations underscore the notion that engagement of non-apoptotic cell death pathways could offer an attractive alternative to the treatment of tumors that have proven refractory to currently employed therapeutic agents.

Necrotic cell death, characterized by plasma membrane rupture (2), has traditionally been considered a non-specific response to acute cellular stress. However, numerous observations over the last decade have revealed that cells can respond to stressful conditions by engaging a variety of pathways that trigger caspase-independent cell death. While these pathways appear distinct and their associated cell death mechanisms go by different names [e.g. necroptosis, ferroptosis, pyroptosis, parthanatos; (4)], their common underlying characteristic is plasma membrane rupture. Thus, our understanding of necrosis has expanded with the realization that it too is a programmed cell death mechanism (5–7). While the therapeutic potential of the various necrotic pathways for cancer remains to be fully explored, recent evidence suggests that engagement of lysosomal cell death (LCD) may offer a particularly attractive avenue.

Lysosomes canonically participate in the digestion of complex molecules such as glycoproteins and glycolipids, recycling basic building blocks such as amino acids and sugars for reuse (8). These organelles are comprised of a limiting lipid bilayer containing numerous structural proteins and channels, an internal glycocalyx lining protecting the limiting membrane from the acidic lysosomal lumen (9), and endosome-derived intraluminal vesicles (ILVs) that harbor enzymes, lipids, and cofactors involved in the highly regulated breakdown of delivered substrates (10, 11). Simultaneously, lysosomes serve as reservoirs for amino acids and Ca^2+^, and engage in nutrient sensing and autophagy (12). However, one of the more underappreciated functions of lysosomes is their role in non-apoptotic cell death, where conditions that promote the breach of the limiting membrane (lysosomal membrane permeabilization, LMP) triggers cascades of events culminating in plasma membrane rupture (13, 14). In this mini-review we discuss LMP and LCD in detail, focusing on agents such as cationic amphiphilic drugs that promote these processes, and highlighting the potential for existing FDA-approved therapeutics to be repurposed for cancer.

## LMP and Its Role in Cancer

Release of cathepsins into the cytosol upon LMP results in the cleavage of multiple proteins, triggering a cascade of events culminating in plasma membrane rupture and LCD (15, 16). This process is akin to caspase-mediated apoptosis following compromise of the mitochondrial outer membrane (17). Interestingly, the degree of lysosomal compromise may dictate the mechanism of cell death; some evidence suggests that extensive LMP can initiate a largely necrotic outcome, while limited LMP can initiate an apoptotic fate (18, 19). As discussed below, a variety of external agents can induce LMP, including lysosomotropic detergents, v-ATPase inhibitors, and cationic amphiphilic drugs [CADs; (20)]. Moreover, LMP efficiency may be influenced by an array of internal factors, including reactive oxygen species (ROS) levels, cytosolic calcium concentration, and the lipid composition of the lysosomal limiting membrane (e.g. cholesterol levels), each of which is commonly dysregulated in cancer (21–23).

Transformation, the process that makes normal cells cancerous, confers marked behavioral changes to the cell, including altered metabolism, enhanced proliferation, increased invasiveness, and drug resistance. These changes are accompanied by dramatic alterations to cellular membranous components, including the cell surface and organelles (3). Increased lysosomal activity is essential to meeting the newly acquired growth demands, and tumor cells often exhibit alterations in lysosomal quantity, volume, membrane composition, hydrolase activity, and energy expended on pH maintenance (20, 21). Paradoxically, the transformation-associated changes critical to efficient tumor cell growth and invasiveness render the cancer cell limiting lysosome membrane more unstable, exposing a cancer-specific vulnerability that may be exploited therapeutically (24, 25). Upon LMP, cathepsins activate various pro-apoptotic proteins including p53, Bid, and TNF (26). However, LCD appears not to rely on p53 or caspases, but instead on ROS and Ca^2+^-dependent calpain proteases (27, 28), providing support for the hypothesis that an LCD-based therapeutic strategy may be exploited in the treatment of tumors resistant to apoptosis-inducing agents.

## LMP Assays

Development of LCD-based therapeutic agents requires robust LMP assays that are sufficiently sensitive to detect low levels or early stages of lysosomal membrane compromise, and are readily adaptable to high throughput formats. Several assays are currently available, each with its strengths and drawbacks.

The LysoTracker probe, available in different colors, accumulates in acidic organelles such as lysosomes where its fluorescence is inversely correlated with pH (29). Thus, LysoTracker fluorescence is diminished as lysosomal pH increases from LMP induction (30), and quantification across dozens to hundreds of untreated versus treated cells can uncover the lysosomal impact of tested agents. However, loss of fluorescence can also reflect the accumulation of drug in lysosomes (31), making interpretations of untested compounds challenging.

A more direct method involves the quantification of fluorescently-tagged dextrans released from lysosomes into the cytosol upon LMP (32). Dextrans are hydrophilic polysaccharides that are endocytosed and delivered to lysosomes following their addition to media of cultured cells. Release of luminal dextrans through lysosomal pores alters fluorescence distribution from a highly punctate to a more diffuse pattern (32, 33). A strength of this method is a range of dextran sizes (10 to 250 kDa) may be employed to estimate the magnitude of drug-induced pores within the membrane (33). A weakness is the dimming of puncta can be difficult to discern at low levels of LMP. However, it has been reported that loss of signal by flow cytometry allows quantification of LMP (34), useful for comparisons across drug candidates.

A similar approach involves the release of cathepsin proteases into the cytosol following LMP. Lysosomal resident cathepsins are canonically involved in the breakdown of proteins, however their cleavage of cytosolic proteins upon LMP is capable of initiating cell death pathways (35). Microscopic analysis of fixed cells with cathepsin antibodies reveals that staining evolves from a highly punctate pattern to a more diffuse pattern with increasing LMP (15). A notable strength of this method is that it can be applied to formalin-fixed, paraffin-embedded tissue samples to assess LMP patient samples and animal models. A variation on this theme assesses cytosolic cathepsin enzyme activity of lysed cells to quantify LMP (32).

Finally, the galectin assay sidesteps issues surrounding the subtle dimming of lysosomal puncta upon LMP, characteristic of the dextran and cathepsin assays, by inverting the strategy to assess increased lysosomal puncta in response to LMP. Galectins are a family of cytosolic and secreted lectins that bind to β-galactoside sugars. Upon LMP, cytosolic lectins diffuse through lysosomal pores and bind to the glycocalyx lining of the inner leaflet of the limiting lysosomal membrane (36); thus, staining of fixed cells with galectin antibodies reveals a more robust punctate pattern after cellular exposure to LMP-inducing agents (32). Galectin abundance in most cells and its immediate translocation to lysosomes make this the most sensitive of LMP assays (37). Moreover, this approach may be coupled with dextran or other lysosomal markers to facilitate high-throughput screening (33).

## LMP-Inducing Agents

In general, three classes of drugs have been demonstrated to induce LMP to provoke lysosomal cell death. The physicochemical properties of lysosomotropic detergents, consisting of a weak base moiety attached to a lipophilic tail, allow these agents to partially permeabilize the limiting lysosomal membrane (38). Their selective accumulation in acidic compartments coupled with the elevated fragility of cancer cell lysosomes relative to non-transformed cells make this class of molecules attractive candidates for the development of novel anti-cancer therapeutics (39). *O*‐methyl‐serine dodecylamine hydrochloride (MSDH), a synthetic detergent under analysis as a potential anticancer therapy, appears to be endocytosed in an inert vesicular form by cells at neutral pH and reconfigures to a toxic micellar form at lysosomal pH (40), suggesting a mechanism by which MSDH may specifically act toward lysosomal membranes and not other membranous structures such as the plasma membrane. L-leucyl-leucine methyl ester (LLOMe), a lysosomotropic agent that provokes the death of cancer cells, also exhibits considerable toxicity toward primary cells (41), highlighting the need for further research into the mechanisms and cell type selectivities of these agents as cancer treatments. Additionally, lysosomotropic detergents have been explored as potential vehicles for directly delivering drugs to lysosomes to induce a more targeted effect (42).

v-ATPase inhibitors block the ATP-dependent proton pump involved in maintaining the cellular pH of lysosomes (20). While inhibition of proton transporters, such as Na^+^/H^+^ exchanger isoform 1 (NHE1), monocarboxylate transporters (MCTs), and carbonic anhydrases (CAs), have been explored in the context of cancer because of dysregulated cytosolic and extracellular hydrogen ion concentrations associated with tumor metabolism (43), a v-ATPase inhibition anti-cancer strategy specifically focuses on disrupting lysosomal pH to provoke LMP. For instance, Bafilomycin A1, a macrolide antibiotic that targets v-ATPase, has exhibited some promise as an antitumor agent (44). Bafilomycin A1 mechanism of action involves the elevation of lysosomal pH and release of cathepsins into the cytoplasm, and has the potential to subvert therapeutic resistance (45). Interestingly, omeprazole, a gastritis and duodenal ulcer treatment targeting the H^+^/K^+^-ATPase of parietal cells through pH modification, has exhibited effectiveness in pancreatic cancer cells by eliciting alterations in lysosomal lipid metabolism to trigger cell death (46). Together, these observations underscore the potential of pH manipulation in the development of novel lysosome-acting therapeutics.

The chemical structure characteristic of CADs, a weak base moiety attached to a hydrophobic region (47), ensure that these molecules accumulate in lysosomes. At neutral pH, the hydrophobic portion permits diffusion across membranes, while at lower pH the base becomes protonated and the charged molecule becomes trapped within the lysosomal lumen (31). CADs are found among a wide variety of drug classes, including antidepressants, neuroleptics, cardiac antiarrhythmics, and tranquilizers (48). Mechanistically, lysosomally trapped CADs are thought to inhibit ILV-localized hydrolytic enzymes to suppress the breakdown of complex lipids (49), which in turn accumulate to levels that compromise lysosomal limiting membrane integrity. For example, siramesine, originally developed as a potential antidepressant, selectively kills cultured cancer cells by inhibiting the lysosomal sphingolipid catabolic enzyme acid sphingomyelinase (ASM) (50–52). Likewise, antihistamines such as loratadine and ebastine exhibit similar efficacy in killing cancer cells through lysosomal membrane destabilization, and epidemiologic evidence points to their effectiveness in reduced cancer mortalities when delivered in conjunction with chemotherapy (53). Accumulating observations suggest that CAD mechanism of action may be particularly well suited to inducing the non-apoptotic death of cancer cells.

## Drug-Induced Phospholipidosis as an Anticancer Strategy

Phospholipidosis results from the excessive accumulation of phospholipids within cells upon CAD treatment (47). As CAD action inhibits breakdown of complex lipids delivered to the lysosome (31, 49), lipid substrates accumulate to form multilamellar bodies reminiscent of membranous cytoplasmic bodies found in lysosomal storage diseases (LSDs) such as Tay-Sachs disease and GM2 gangliosidoses (54). LSDs encompass over 70 very rare genetic diseases that arise from deleterious mutations in genes responsible for lysosomal function and homeostasis (55). While these diseases have dire human health consequences (55), often leading to death within the second or third decade, harnessing the ability of drugs to induce a transient but acutely lipidotic state to specifically kill cancer cells offers tremendous therapeutic potential for tumors refractory to other treatment options. It is important to note that clinical studies suggest CAD-induced phospholipidosis in normal tissues is reversible with drug withdrawal (54), so adverse side effects may be rapidly ameliorated.

The primary consideration in cancer therapeutic development is the ability of drug to reach its tumor tissue and molecular target at sufficient concentrations to elicit a pharmacologic effect, while minimizing the impact at normal tissues. Certainly, the intrinsic ability of CADs to concentrate in lysosomes, the site of their molecular target, facilitates efficacy, and the abundance and lability of transformed cell lysosomes relative to those of normal cells minimizes off-target concerns. At the same time, CADs tend to accumulate within tumors relative to normal tissue because of their lower cytosolic pH and the lower difference between cytosolic and lysosomal pH (56, 57). On the other hand, CADs also distribute efficiently to tissue types rich in lysosomes, including lung, liver, and kidney (56), suggesting these sites may be most susceptible to CAD-induced side effects.

Siramesine and hexamethylene amiloride (HMA) are examples of CADs that illustrate the potential of the phospholipidosis induction strategy in cancer therapy. As mentioned above, siramesine specifically targets a variety of cancer cell lines through ASM inhibition (50, 51), and its mechanism of action appears to involve inhibition of ASM binding to and activation by the acidic lysosome-specific lipid bis(monoacylglycero)phosphate (BMP) within ILVs (51). Lysosomal accumulation of ASM substrates upon drug treatment may contribute to membrane destabilization and LMP. Interestingly, altered sphingomyelin metabolism common to tumors may further sensitize cells to siramesine, and evidence has been presented that siramesine can reverse drug resistance to confer tumor cell sensitivity to conventional chemotherapeutics (51), underscoring the potential of this drug as a repurposed anticancer therapeutic. HMA, a derivative of the diuretic amiloride that has been used clinically for over 50 years, is cytotoxic toward breast cancer cells independent of tumor subtype or species, while exhibiting marginal impact on non-transformed cells from a variety of tissues (58). Notably, HMA cytotoxicity contrasts with that of conventional chemotherapeutics in that it kills tumor cells trapped in G1 phase of the cell cycle, suggesting that poorly proliferative tumor cell populations resistant to traditional chemotherapeutics are susceptible to CADs. Mechanistically, HMA induces the formation of multilamellar bodies in lysosomes of treated tumor cells upon very short (1–3 h) exposure, and triggers a caspase-independent and cathepsin-, Ca^2+^- and ROS-dependent cell death mechanism within 24 hours (58). Further studies assessing HMA-induced lysosomal lipid metabolism are warranted.

## Non-CAD Induction of LCD

Though CADs offer a straightforward approach to engaging LCD to combat cancer, other agents and strategies also show significant promise. In addition to the membrane-permeabilizing lysosomotropic detergents and pH modifying v-ATPase inhibitors discussed above, agents that interfere with lysosomal iron disposition are also attractive candidate LCD inducers. As a primary store of cellular iron, acute dysregulation of lysosomal iron homeostasis triggers the ferroptosis cell death mechanism (4, 59, 60). Salinomycin, an antimicrobial used to treat coccidiosis, and its derivative ironomycin have exhibited anti-cancer effects, notably toward cancer stem cells (61, 62). Their mechanism of action appears to involve the sequestration of iron within the lysosome, leading to the production of high levels of ROS that destabilize the limiting membrane, induce LMP, and ultimately necrotic cell death (63).

## Conclusions

**Figure 1:**
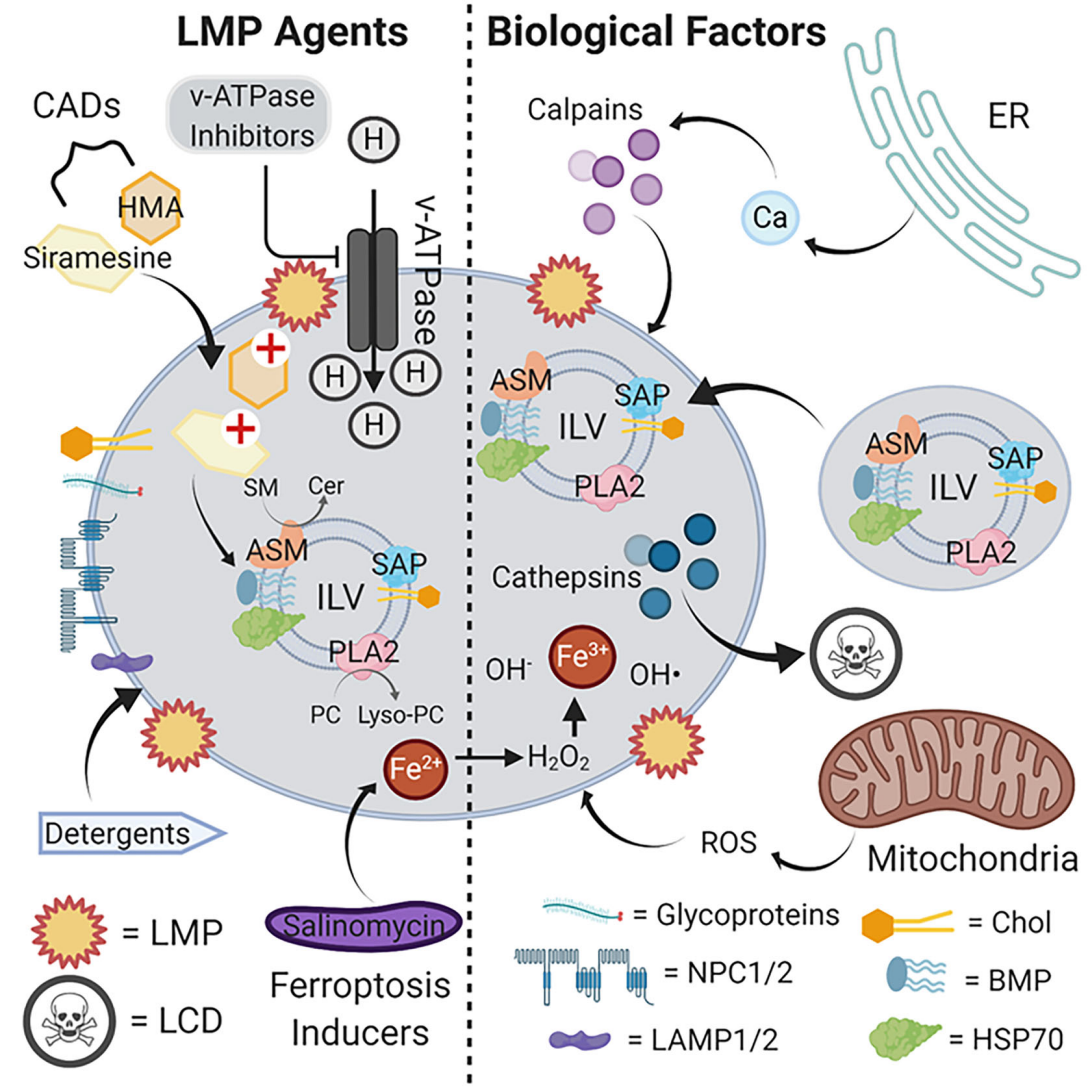
LMP agents and biological factors that induce lysosomal rupture and cell death. The limiting lysosomal membrane employs various glycoproteins, NPC1/2 and LAMP1/2, and a characteristic lipid composition to maintain its functional integrity as a barrier with the cytosol. Internally, ILVs delivered from endosomes *via* vesicles bind to and activate lysosomal hydrolases responsible for the metabolic breakdown of delivered glycolipids to maintain cellular steady-state levels. CADs such as siramesine and HMA compromise this metabolic pathway, likely by interfering with the activation of enzymes such as ASM by ILV-localized acidic lipids such as BMP. The resulting accumulation of glycolipid substrates such as SM leads to limiting membrane destabilization, LMP and cathepsin release, and ultimately cell death. By suppressing proton import, v-ATPase inhibitors similarly suppress lysosomal hydrolase function by elevating luminal pH beyond the optimum for catalytic activity. Lysosomotropic detergents directly disrupt the limiting membrane by partially solubilizing lipid components, and ferroptosis inducers such as salinomycin sequester iron in lysosomes to promote ROS accumulation and limiting membrane destabilization through lipid oxidation. Internally-produced ROS from mitochondria can also contribute to limiting membrane destabilization, as can the release of Ca^2+^ from intracellular stores such as the ER to activate calpains. Abbreviations: ASM, acid sphingomyelin; BMP, bis(monoacylglycero)phosphate; CADs, cationic amphiphilic drugs; Cer, ceramides; Chol, cholesterol; ER, endoplasmic reticulum; HSP70, heat shock protein 70; HMA, hexamethylene amiloride; ILV, intraluminal vesicle; LAMP1/2, lysosomal-associated membrane protein 1/2; LCD, lysosomal cell death; LMP, lysosomal membrane permeabilization; lyso-PC, lysophosphatidylcholine; NPC1/2, Niemann-Pick disease 1/2; PC, phosphatidylcholine; PLA2, phospholipase 2; ROS, reactive oxygen species; SAP, saposins; SM, sphingomyelins. Illustration created using Biorender.

Lysosomes are powerful organelles that maintain steady-state levels of a variety of cellular metabolites by mediating their breakdown upon delivery. Acute disruption of these homeostatic processes can lead to LMP, which ultimately engages LCD programmed necrotic cell death. **Figure 1** summarizes the biological and chemical factors implicated in triggering LMP, highlighting the therapeutic potential of repurposed CADs, v-ATPase inhibitors, lysosomotropic detergents, and ferroptosis inducers. Although underappreciated in the cancer therapeutics field, accumulating studies point to exploitation of this mechanism for tremendous potential in targeting apoptosis-resistant tumor cell subpopulations. Advantages include the selective action of LCD inducers toward tumor versus non-transformed cells, minimizing side effects, and the ability of LCD inducers to trigger death in quiescent cell populations resistant to conventional chemotherapeutics.

A significant consideration in cancer therapeutic development concerns the effective integration of a novel drug with existing treatment paradigms. A popular approach is to incorporate the newly developed drug into current standard-of-care treatment protocols, with the hope of realizing synergistic efficacies with added agents. Such an approach could prove particularly effective with LMP-inducing agents. It has been suggested that lysosomes contribute to drug resistance by sequestering chemotherapeutics (doxorubicin, mitoxantrone, sunitinib, etc.), decreasing their availability and effective concentrations at target sites (64); thus, the abrupt release of stored chemotherapeutics into the cytosol after CAD treatment could allow for a potent one-two punch (65). On the other hand, doubling up on therapeutic agents runs the risk of developing synergistic toxicities and unwanted off-target effects. Given that LCD-inducing agents uniquely target quiescent and apoptosis- and therapy-resistant cell populations, a more fruitful strategy may involve delivery of such agents following standard-of-care treatment to eradicate remaining tumor cells and minimize chances for recurrence.

Going forward, LMP screens will identify novel agents that might be developed into more effective anti-cancer therapeutics. This is somewhat ironic in that the phospholipidosis side effect of CADs has been known for decades, and many investigators omit compounds from screens for drugs for other disease states whose structures might provoke such a phenotype. Deeper analysis will uncover the specific mechanisms by which CADs act. In this regard, it is important to note that different CADs exhibit somewhat different phenotypes and cytotoxic parameters, not surprising given that the phenotypes of lysosomal storage diseases can differ substantially. It is likely that different CADs preferentially target different enzymes of lysosome metabolism. As the molecular targets of CADs are uncovered and their structures elucidated, rational drug design approaches may be utilized to develop inhibitors; conferring CAD characteristics to lysosomal enzyme inhibitors of moderate efficiency could markedly enhance their potency in cells by promoting their lysosomal accumulation. These approaches will likely take years to decades to fully unfold. In the meantime, efforts to repurpose roughly six dozen existing clinically-employed CAD molecules to anti-cancer purposes could give us a substantial leg up on the LCD approach.

## Author Contributions

MH and KC contributed to the research for and the writing of the manuscript. All authors contributed to the article and approved the submitted version.

## Funding

The authors are supported by NIH grants CA230742 and CA250211.

## Conflict of Interest

The authors declare that the research was conducted in the absence of any commercial or financial relationships that could be construed as a potential conflict of interest.

## References

[1] Siegel RL Miller KD Jemal A Cancer statistics, 2020. CA Cancer J Clin (2020) 70:7–30. 10.3322/caac.21590 31912902

[2] Green DR Llambi F Cell Death Signaling. Cold Spring Harb Perspect Biol (2015) 7(12):27–51. 10.1101/cshperspect.a006080 PMC4665079 26626938

[3] Hanahan D Weinberg RA Hallmarks of cancer: the next generation. Cell (2011) 144:646–74. 10.1016/j.cell.2011.02.013 21376230

[4] Galluzzi L Vitale I Aaronson SA Abrams JM Adam D Agostinis P Molecular mechanisms of cell death: recommendations of the Nomenclature Committee on Cell Death 2018. Cell Death Differ (2018) 25:486–541. 10.1038/s41418-018-0102-y 29362479 PMC5864239

[5] Vanden Berghe T Linkermann A Jouan-Lanhouet S Walczak H Vandenabeele P Regulated necrosis: the expanding network of non-apoptotic cell death pathways. Nat Rev Mol Cell Biol (2014) 15:135–47. 10.1038/nrm3737 24452471

[6] Tait SWG Ichim G Green DR Die another way–non-apoptotic mechanisms of cell death. J Cell Sci (2014) 127:2135–44. 10.1242/jcs.093575 PMC4021468 24833670

[7] Leist M Jäättelä M Four deaths and a funeral: from caspases to alternative mechanisms. Nat Rev Mol Cell Biol (2001) 2:589–98. 10.1038/35085008 11483992

[8] de Duve C Lysosomes revisited. Eur J Biochem (1983) 137:391–7. 10.1111/j.1432-1033.1983.tb07841.x 6319122

[9] Settembre C Fraldi A Medina DL Ballabio A Signals from the lysosome: a control centre for cellular clearance and energy metabolism. Nat Rev Mol Cell Biol (2013) 14:283–96. 10.1038/nrm3565 PMC4387238 23609508

[10] Piper RC Katzmann DJ Biogenesis and function of multivesicular bodies. Annu Rev Cell Dev Biol (2007) 23:519–47. 10.1146/annurev.cellbio.23.090506.123319 PMC2911632 17506697

[11] Bonifacino JS Neefjes J Moving and positioning the endolysosomal system. Curr Opin Cell Biol (2017) 47:1–8. 10.1016/j.ceb.2017.01.008 28231489 PMC5537022

[12] Todkar K Ilamathi HS Germain M Mitochondria and Lysosomes: Discovering Bonds. Front Cell Dev Biol (2017) 5:106. 10.3389/fcell.2017.00106 29270406 PMC5725469

[13] Mukhopadhyay S Panda PK Sinha N Das DN Bhutia SK Autophagy and apoptosis: where do they meet? Apoptosis (2014) 19:555–66. 10.1007/s10495-014-0967-2 24415198

[14] Galluzzi L Bravo-San Pedro JM Kroemer G Organelle-specific initiation of cell death. Nat Cell Biol (2014) 16:728–36. 10.1038/ncb3005 25082195

[15] Wang F Gómez-Sintes R Boya P Lysosomal membrane permeabilization and cell death. Traffic (2018) 19:918–31. 10.1111/tra.12613 30125440

[16] Repnik U Stoka V Turk V Turk B Lysosomes and lysosomal cathepsins in cell death. Biochim Biophys Acta (2012) 1824:22–33. 10.1016/j.bbapap.2011.08.016 21914490

[17] Tait SWG Green DR Mitochondrial regulation of cell death. Cold Spring Harb Perspect Biol (2013) 5(9):130–45. 10.1101/cshperspect.a008706 PMC3753705 24003207

[18] Kirkegaard T Jäättelä M Lysosomal involvement in cell death and cancer. Biochim Biophys Acta (2009) 1793:746–54. 10.1016/j.bbamcr.2008.09.008 18948147

[19] Aits S Jäättelä M Lysosomal cell death at a glance. J Cell Sci (2013) 126:1905–12. 10.1242/jcs.091181 23720375

[20] Domagala A Fidyt K Bobrowicz M Stachura J Szczygiel K Firczuk M Typical and Atypical Inducers of Lysosomal Cell Death: A Promising Anticancer Strategy. Int J Mol Sci (2018) 19(8):2256–72. 10.3390/ijms19082256 PMC6121368 30071644

[21] Serrano-Puebla A Boya P Lysosomal membrane permeabilization as a cell death mechanism in cancer cells. Biochem Soc Trans (2018) 46:207–15. 10.1042/BST20170130 29472365

[22] Görlach A Bertram K Hudecova S Krizanova O Calcium and ROS: A mutual interplay. Redox Biol (2015) 6:260–71. 10.1016/j.redox.2015.08.010 PMC4556774 26296072

[23] Boya P Kroemer G Lysosomal membrane permeabilization in cell death. Oncogene (2008) 27:6434–51. 10.1038/onc.2008.310 18955971

[24] Kallunki T Olsen OD Jäättelä M Cancer-associated lysosomal changes: friends or foes? Oncogene (2013) 32:1995–2004. 10.1038/onc.2012.292 22777359

[25] Fehrenbacher N Jäättelä M Lysosomes as targets for cancer therapy. Cancer Res (2005) 65:2993–5. 10.1158/0008-5472.CAN-05-0476 15833821

[26] Halaby R Role of lysosomes in cancer therapy. Res Rep Biol (2015) 6:147–55. 10.2147/RRB.S83999

[27] Appelqvist H Wäster P Kågedal K Öllinger K The lysosome: from waste bag to potential therapeutic target. J Mol Cell Biol (2013) 5:214–26. 10.1093/jmcb/mjt022 23918283

[28] Repnik U Hafner Česen M Turk B Lysosomal membrane permeabilization in cell death: concepts and challenges. Mitochondrion (2014) 19:49–57. 10.1016/j.mito.2014.06.006 24984038

[29] Chazotte B Labeling lysosomes in live cells with LysoTracker. Cold Spring Harb Protoc (2011) 2011:pdb.prot5571. 10.1101/pdb.prot5571 21285271

[30] Johnson DE Ostrowski P Jaumouillé V Grinstein S The position of lysosomes within the cell determines their luminal pH. J Cell Biol (2016) 212:677–92. 10.1083/jcb.201507112 PMC4792074 26975849

[31] Kazmi F Hensley T Pope C Funk RS Loewen GJ Buckley DB Lysosomal sequestration (trapping) of lipophilic amine (cationic amphiphilic) drugs in immortalized human hepatocytes (Fa2N-4 cells). Drug Metab Dispos (2013) 41:897–905. 10.1124/dmd.112.050054 23378628 PMC3608459

[32] Aits S Jäättelä M Nylandsted J Chapter 13 - Methods for the quantification of lysosomal membrane permeabilization: A hallmark of lysosomal cell death. In: Platt F Platt N, editors. Methods in Cell Biology. Cambridge, MA: Academic Press (2015). p. 261–85. 10.1016/bs.mcb.2014.10.032 PMC7611294 25665450

[33] Aits S Methods to Detect Loss of Lysosomal Membrane Integrity. In: Ktistakis N Florey O, editors. Autophagy: Methods and Protocols. New York, NY: Springer New York (2019). p. 315–29. 10.1007/978-1-4939-8873-0_21 30610707

[34] Eriksson I Öllinger K Appelqvist H Öllinger K Appelqvist H, editors. Lysosomes: Methods and Protocols. New York, NY: Springer New York (2017). p. 179–89. 10.1007/978-1-4939-6934-0_11

[35] Turk V Stoka V Vasiljeva O Renko M Sun T Turk B Cysteine cathepsins: from structure, function and regulation to new frontiers. Biochim Biophys Acta (2012) 1824:68–88. 10.1016/j.bbapap.2011.10.002 22024571 PMC7105208

[36] Jia J Abudu YP Claude-Taupin A Gu Y Kumar S Choi SW Galectins Control mTOR in Response to Endomembrane Damage. Mol Cell (2018) 70:120–35. 10.1016/j.molcel.2018.03.009 PMC5911935 29625033

[37] Aits S Kricker J Liu B Ellegaard A-M Hämälistö S Tvingsholm S Sensitive detection of lysosomal membrane permeabilization by lysosomal galectin puncta assay. Autophagy (2015) 11:1408–24. 10.1080/15548627.2015.1063871 PMC4590643 26114578

[38] Villamil Giraldo AM Appelqvist H Ederth T Öllinger K Lysosomotropic agents: impact on lysosomal membrane permeabilization and cell death. Biochem Soc Trans (2014) 42:1460–4. 10.1042/BST20140145 25233432

[39] Firestone RA Pisano JM Bonney RJ Lysosomotropic agents. 1. Synthesis and cytotoxic action of lysosomotropic detergents. J Med Chem (1979) 22:1130–3. 10.1021/jm00195a026 114658

[40] Villamil Giraldo A-M Eriksson I Wennmalm S Fyrner T Ederth T Öllinger K Interactions of the Lysosomotropic Detergent O-Methyl-Serine Dodecylamide Hydrochloride (MSDH) with Lipid Bilayer Membranes-Implications for Cell Toxicity. Int J Mol Sci (2020) 21:3136. 10.3390/ijms21093136 PMC7247706 32365555

[41] Kavčič N Butinar M Sobotič B Hafner Česen M Petelin A Bojić L Intracellular cathepsin C levels determine sensitivity of cells to leucyl-leucine methyl ester-triggered apoptosis. FEBS J (2020). 10.1111/febs.15326 32319717

[42] Villamil Giraldo AM Fyrner T Wennmalm S Parikh AN Öllinger K Ederth T Spontaneous Vesiculation and pH-Induced Disassembly of a Lysosomotropic Detergent: Impacts on Lysosomotropism and Lysosomal Delivery. Langmuir (2016) 32:13566–75. 10.1021/acs.langmuir.6b03458 27936755

[43] Harguindey S Arranz JL Wahl ML Orive G Reshkin SJ Proton transport inhibitors as potentially selective anticancer drugs. Anticancer Res (2009) 29:2127–36. 19528473

[44] Bowman EJ Gustafson KR Bowman BJ Boyd MR Identification of a new chondropsin class of antitumor compound that selectively inhibits V-ATPases. J Biol Chem (2003) 278:44147–52. 10.1074/jbc.M306595200 12944415

[45] Nakashima S Hiraku Y Tada-Oikawa S Hishita T Gabazza EC Tamaki S Vacuolar H+-ATPase inhibitor induces apoptosis via lysosomal dysfunction in the human gastric cancer cell line MKN-1. J Biochem (2003) 134:359–64. 10.1093/jb/mvg153 14561721

[46] Udelnow A Kreyes A Ellinger S Landfester K Walther P Klapperstueck T Omeprazole inhibits proliferation and modulates autophagy in pancreatic cancer cells. PloS One (2011) 6:e20143. 10.1371/journal.pone.0020143 21629657 PMC3101238

[47] Halliwell WH Cationic amphiphilic drug-induced phospholipidosis. Toxicol Pathol (1997) 25:53–60. 10.1177/019262339702500111 9061852

[48] Vater M Möckl L Gormanns V Schultz Fademrecht C Mallmann AM Ziegart-Sadowska K New insights into the intracellular distribution pattern of cationic amphiphilic drugs. Sci Rep (2017) 7:44277. 10.1038/srep44277 28281674 PMC5345070

[49] Gulbins E Kolesnick RN It takes a CAD to kill a tumor cell with a LMP. Cancer Cell (2013) 24:279–81. 10.1016/j.ccr.2013.08.025 24029224

[50] Ostenfeld MS Fehrenbacher N Høyer-Hansen M Thomsen C Farkas T Jäättelä M Effective tumor cell death by sigma-2 receptor ligand siramesine involves lysosomal leakage and oxidative stress. Cancer Res (2005) 65:8975–83. 10.1158/0008-5472.CAN-05-0269 16204071

[51] Petersen NHT Olsen OD Groth-Pedersen L Ellegaard A-M Bilgin M Redmer S Transformation-associated changes in sphingolipid metabolism sensitize cells to lysosomal cell death induced by inhibitors of acid sphingomyelinase. Cancer Cell (2013) 24:379–93. 10.1016/j.ccr.2013.08.003 24029234

[52] Beckmann N Sharma D Gulbins E Becker KA Edelmann B Inhibition of acid sphingomyelinase by tricyclic antidepressants and analogons. Front Physiol (2014) 5:331. 10.3389/fphys.2014.00331 25228885 PMC4151525

[53] Ellegaard A-M Dehlendorff C Vind AC Anand A Cederkvist L Petersen NHT Repurposing Cationic Amphiphilic Antihistamines for Cancer Treatment. EBioMedicine (2016) 9:130–9. 10.1016/j.ebiom.2016.06.013 PMC4972561 27333030

[54] Breiden B Sandhoff K Emerging mechanisms of drug-induced phospholipidosis. Biol Chem (2019) 401:31–46. 10.1515/hsz-2019-0270 31408430

[55] Platt FM Emptying the stores: lysosomal diseases and therapeutic strategies. Nat Rev Drug Discov (2018) 17:133–50. 10.1038/nrd.2017.214 29147032

[56] Kaufmann AM Krise JP Lysosomal sequestration of amine-containing drugs: analysis and therapeutic implications. J Pharm Sci (2007) 96:729–46. 10.1002/jps.20792 17117426

[57] Zhitomirsky B Assaraf YG The role of cytoplasmic-to-lysosomal pH gradient in hydrophobic weak base drug sequestration in lysosomes. Cancer Cell Microenviron (2015) 2:2. 10.3390/ijms21124392

[58] Rowson-Hodel AR Berg AL Wald JH Hatakeyama J VanderVorst K Curiel DA Hexamethylene amiloride engages a novel reactive oxygen species- and lysosome-dependent programmed necrotic mechanism to selectively target breast cancer cells. Cancer Lett (2016) 375:62–72. 10.1016/j.canlet.2016.02.042 26944316 PMC5554595

[59] Dielschneider RF Henson ES Gibson SB Lysosomes as Oxidative Targets for Cancer Therapy. Oxid Med Cell Longev (2017) 2017:3749157. 10.1155/2017/3749157 28757908 PMC5516749

[60] Li J Cao F Yin H-L Huang Z-J Lin Z-T Mao N Ferroptosis: past, present and future. Cell Death Dis (2020) 11:88. 10.1038/s41419-020-2298-2 32015325 PMC6997353

[61] Gupta PB Onder TT Jiang G Tao K Kuperwasser C Weinberg RA Identification of selective inhibitors of cancer stem cells by high-throughput screening. Cell (2009) 138:645–59. 10.1016/j.cell.2009.06.034 PMC4892125 19682730

[62] Zhao B Li X Wang Y Shang P Iron-dependent cell death as executioner of cancer stem cells. J Exp Clin Cancer Res (2018) 37:79. 10.1186/s13046-018-0733-3 29636068 PMC5894200

[63] Mai TT Hamaï A Hienzsch A Cañeque T Müller S Wicinski J Salinomycin kills cancer stem cells by sequestering iron in lysosomes. Nat Chem (2017) 9:1025–33. 10.1038/nchem.2778 PMC5890907 28937680

[64] Zhitomirsky B Assaraf YG Lysosomal sequestration of hydrophobic weak base chemotherapeutics triggers lysosomal biogenesis and lysosome-dependent cancer multidrug resistance. Oncotarget (2015) 6:1143–56. 10.18632/oncotarget.2732 PMC4359223 25544758

[65] Groth-Pedersen L Jäättelä M Combating apoptosis and multidrug resistant cancers by targeting lysosomes. Cancer Lett (2013) 332:265–74. 10.1016/j.canlet.2010.05.021 20598437

